# Supplementary material for: A Global Screen for Assembly State Changes of the Mitotic Proteome by SEC-SWATH-MS
Source: Cell Syst. 2020 Feb 26;10(2):133–155.e6. doi: 10.1016/j.cels.2020.01.001 (PMC7042714; doi:10.1016/j.cels.2020.01.001)

Q03518 | TAP1_HUMAN | TAP1 ABCB2 PSF1 RING4 Y3
Monomer MW [kDa]: 87.218 Monomer expected elution fraction: 41

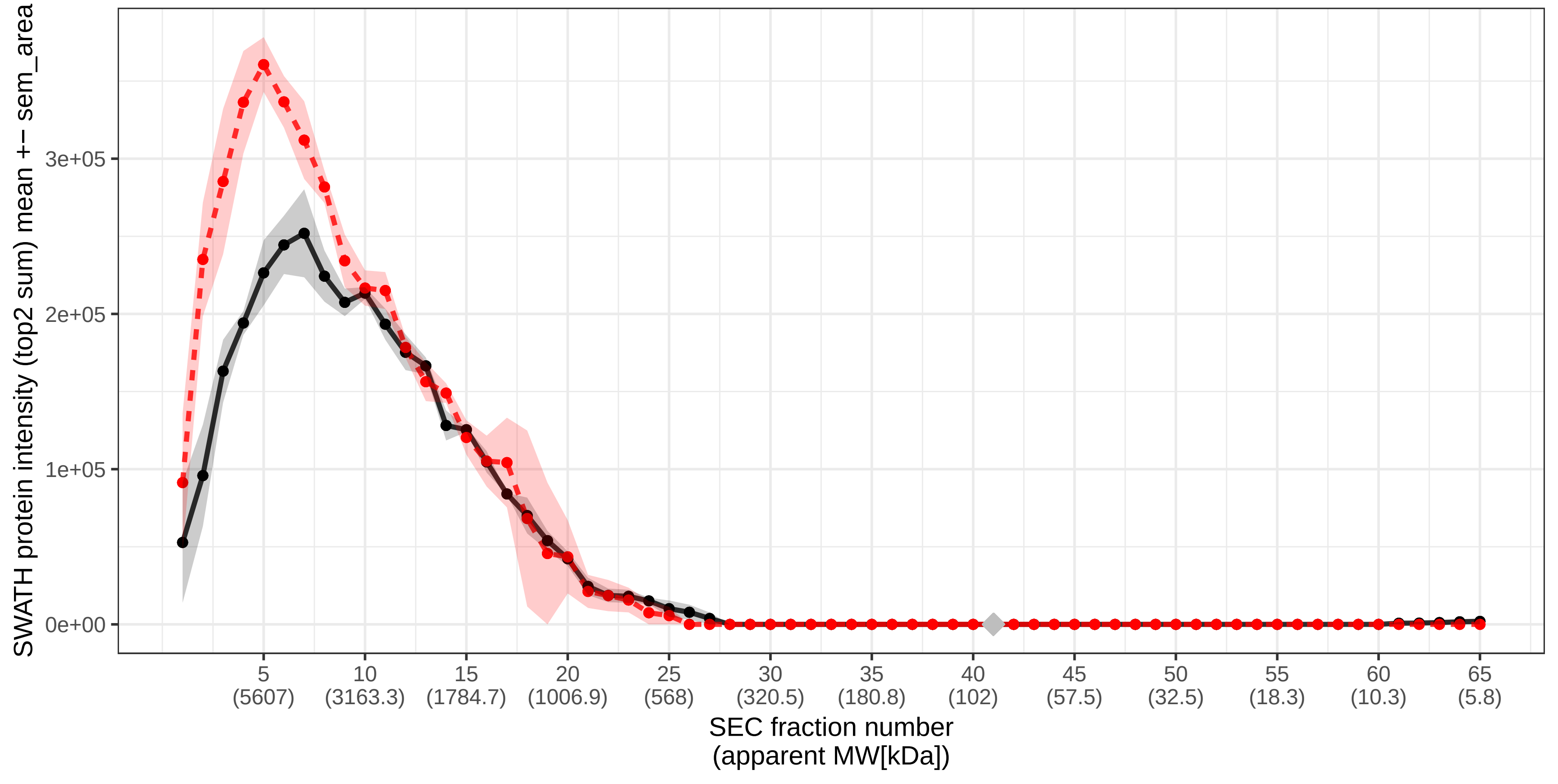

Supplement: Data S1. SEC-SWATH-MS Protein Chromatograms, Related to Figure 1 [file mmc6.zip › SECchrom_Q03518_TAP1_HUMAN_TAP1_ABCB2_PSF1_RING4_Y3.pdf]
